# Differences in Metabolites of Different Tongue Coatings in Patients with Chronic Hepatitis B

**DOI:** 10.1155/2013/204908

**Published:** 2013-04-17

**Authors:** Yu Zhao, Xiao-jun Gou, Jian-ye Dai, Jing-hua Peng, Qin Feng, Shu-jun Sun, Hui-juan Cao, Ning-ning Zheng, Jun-wei Fang, Jian Jiang, Shi-bing Su, Ping Liu, Yi-yang Hu, Yong-yu Zhang

**Affiliations:** ^1^Institute of Liver Disease, Shuguang Hospital, Shanghai University of Traditional Chinese Medicine, 258 Zhangheng Road, Pudong District, Shanghai 201203, China; ^2^Center for Traditional Chinese Medicine and Systems Biology of Shanghai University of Traditional Chinese Medicine, 1200 Cailun Road, Pudong District, Shanghai 201203, China; ^3^E-Institute of Shanghai Municipal Education Commission, Shanghai 201203, China

## Abstract

Tongue coating is one of the important foundations of tongue diagnosis in traditional Chinese medicine (TCM) and plays an important role in reflecting the occurrence, development, and prognosis of the disease. However, its material basis is still poorly understood. In this study, a urinary metabonomic method based on gas chromatography coupled to mass spectrometry (GC/MS) was developed. The distinct clustering in metabolic profile was observed from Group A (thick yellow coating in patients with chronic hepatitis B), Group B (thick white coating in patients with chronic hepatitis B), and Group C (thin white coating with healthy humans) using orthogonal projections to latent structures (OPLS). Based on the variable of importance in the project (VIP) values, some significantly changed metabolites have been identified. These changes were related to the disturbance in energy metabolism, amino acid metabolism, nucleotide metabolism, and gut microflora, which were helpful to understand the material basis leading to the formation of tongue coating. This study demonstrated that tongue coating may have an objective material basis.

## 1. Introduction

Tongue coating is one of the direct objective foundations for TCM clinical diagnosis and treatment, so it is of great importance for syndrome differentiation, determining the treating principle, prescribing a formula, and predicting the prognosis [1]. TCM holds that the tongue coating is formed by the evaporation of stomach qi and is closely related to the conditions of the body organs function, qi, and blood [2, 3]. Organ conditions, properties, and variations of pathogens can be revealed through observation of tongue [4]. As is stated in Danxi's Experience on Medicine (Danxi-XinFa), “To want knowing the internal conditions of the body, the external manifestations of the body should be observed, to diagnose the external parts of the body may also know the internal conditions. That is because the internal conditions of the body are always reflected on the exterior of the body” [5]. Therefore, based on the above analysis, we speculated that the body of the patient with the appearing tongue coating in TCM has the material basis that triggers a series of symptoms and reasoned that some changes of the material basis led to alterations of the tongue coating in TCM.

In recent years, many scholars have made certain achievements on the tongue coating's research by means of new research methods. Deng et al. [6] carried out the comparative study on normal tongue coating of primary liver cancer patients and healthy people using the tip of the tongue microcirculation inspection and tongue coating exfoliated cells staining, which displayed that the total score of microcirculation of tongue tip and the maturation index of exfoliated cells from tongue coating were both higher in patients with primary liver cancer than healthy adults with normal tongue manifestation. Another study showed that tongue epithelial apoptosis was significantly reduced, and the total number of bacteria was obviously increased in yellow greasy coating of dampness-heat syndrome with spleen and stomach patients than those in normal tongue of healthy adults [7]. Available literature had reviewed that reproductive activity enhancement of glossal epithelial cells was one of the main characteristics of thick greasy tongue fur formation, an increase in vasopermeability was closely associated with thick greasy tongue fur formation, and tight junction structural variation of vascular endothelial cells might play an important role in the pathological and physiological process of thick greasy tongue fur formation [8]. Jiang et al. [9] identified 715 differentially abundant, species-level operational taxonomic units (OUTs) on tongue coatings of the enrolled patients compared to healthy controls using next-generation sequencing on tongue coating samples, further identified two subtypes of tongue coating microbiomes, and detected 123 “Cold OTUs” and 258 “Hot OTUs” that were enriched in patients with Cold Syndrome and Hot Syndrome representing the “Cold Microbiota” and “Hot Microbiota,” respectively, which illustrated the potential of the tongue-coating microbiome as a novel holistic biomarker for characterizing patient subtypes, et al. These studies have explored some mechanisms of the tongue coating's formation in terms of morphology, cellular and molecular biology, and microecology, which played a big role in revealing the tongue material foundation. However, current research is just limited to studying tongue coating's principle from known biological function indicators based on the tongue itself. It is one of the more important scientific issues in studying the relationship between tongue coating and the whole inner environment, which is helpful to reveal the biological basis of tongue coating in the overall level. Urine is the ideal window used to understand human physiological and pathological states. Changes of the metabolites in the urine are closely related to the inner environment. Tongue coating is connected with the organs, meridians, qi, blood, and body fluid. Changes of tongue coating can't be separated from the inner environment of the body. The tongue coating is also an important window to the reaction of physiological and pathological changes. Therefore, studying metabolites in the urine is another way to understand the material basis of tongue coating and might be a promising contribution to tongue diagnosis.

Metabonomics is an important branch of systems biology. It is defined as “the quantitative measurement of the dynamic multiparametric metabolic response of living systems to pathophysiologic stimuli or genetic modification” [10]. It is based on the analysis of the endogenous metabolites of various biofluids and tissues extractions and aims to harvest a latent relationship between the changed metabolic profiles and the physiological status of the biosystem [11]. This research strategy agrees with the integrity and systemic feature of TCM [12]. Metabonomic technique has shown potential and has been applied in TCM fields [13], such as syndrome research [14], assessing therapeutic effects of Chinese medicine [15], and evaluation of Chinese medicine toxicity [16]. However, there were few reports on exploring the material basis of the tongue coating in TCM with metabonomic approach. In the present study, we collected urine samples from thick yellow coating and thick white coating in patients with chronic hepatitis B, and thin white coating with healthy controls, respectively. We investigated the urine metabolite variation between the different tongue coatings in TCM using metabonomic method based on GC/MS. Meanwhile, we used this approach in an attempt to explore the possible material basis of tongue coating.

## 2. Materials and Methods

### 2.1. Chemicals

Heptanes and methanol were analytical grade and obtained from China National Pharmaceutical Group Corporation (Shanghai, China). Myristic acid, urease, N, O-bis (trimethylsilyl)trifluoroacetamide, and methoxyamine hydrochloride were obtained from Sigma-Aldrich (St. Louis, MO, USA). 

### 2.2. Study Subjects

**Figure 1:**
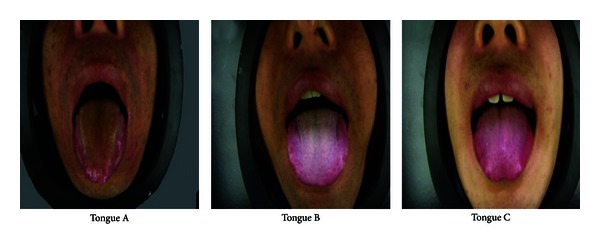
Samples of the different tongue coatings. Tongue A: thick yellow coating, tongue B: thick white coating, and tongue C: thin white coating.

From July 2009 to October 2010, 100 patients with chronic hepatitis B (50 with thick yellow coating and 50 with thick white coating) were recruited from the Department of Hepatitis of Shuguang Hospital Affiliated to Shanghai University of Traditional Chinese Medicine, and 25 healthy people were recruited as the normal group (normal tongue coating : thin white coating). They were provided written informed consent under the guideline approved by the Ethics Committee of the Institute of Shuguang Hospital Affiliated to Shanghai University of TCM. The diagnostic criteria for chronic hepatitis B was based on “*the guideline of prevention and treatment for chronic hepatitis B*” formulated by Chinese Society of Hepatology and Chinese Society of Infectious Diseases, Chinese Medical Association [17]. The different tongue coatings in TCM, including thick yellow coating, thick white coating, and thin white coating, were differentiated according to “*Diagnostics of Tradition Chinese Medicine*” [18] (Figure 1). Patients suffering from other serious diseases involving alcoholic liver disease, autoimmune liver disease and other liver diseases, cirrhosis, or suspected hepatocellular carcinoma were excluded from the study. Moreover, pregnancy or breast-feeding women were also excluded.

The subjects were divided into three groups according to their tongue coatings in TCM, including thick yellow coating (Group A), thick white coating (Group B), and thin white coating (Group C).

### 2.3. Tongue Coating Diagnosis

We formulated the tongue states table based on the contents of tongue diagnosis according to “*Diagnostics of Tradition Chinese Medicine*,” including inspection of the tongue body (tongue color, tongue shape, and tongue movement), inspection of tongue coating (thickness, moistening and dryness of the tongue coating, and greasy and putrid tongue coating), and the colors of tongue coating (white tongue coating, yellow tongue coating, and grayish black tongue coating).

The tongue states in all subjects were observed and recorded by two certified and experienced TCM doctors and then photographed by a professional tongue image instrument (DAOSH, ZBOX); tongue images were fed into the computer. Tongue coating images were analyzed objectively by three experts from the same department in each site, who were experienced physicians and well trained in standard operation procedures. Diagnostic information of the tongue coating was determined independently by three experts to ensure an objective evaluation. If the three were consistent, the subject would be included in the study; otherwise, he/she will be excluded. 

### 2.4. Clinical and Laboratory Assessment

At the same time, clinical data including age, sex, and course of disease were collected by a senior physician. Serum levels of total bilirubin (TBIL), direct bilirubin (DBIL), indirect bilirubin (IDBIL), alanine aminotransferase (ALT), aspartate aminotransferase (AST), gamma glutamyl transferase (GGT), alkaline phosphatase (ALP), total protein (TP), albumin (ALB), total bile acid (TBA), HBsAg, HBeAg, and DNA were measured in Shuguang Hospital.

### 2.5. Sample Collection

Subjects were required to pay attention to a light diet before the collection to avoid an interference with human metabolism. All collected urine samples (12 h) in a fasting condition were immediately stored at −80°C after centrifugation at 3000 rpm for 10 min to remove residues. Blood samples were obtained from the venous blood, centrifuged at 3000 rpm for 10 min and stored at −80°C for hepatic function analysis.

### 2.6. Urine Sample Preparation

The urine samples were prepared according to published methods with minor modifications [19]. Two hundred microliters of urine added with 30 units of urease was incubated at 37°C for 15 min. Then, 800 *μ*L of methanol and 10 *μ*L of myristic acid (1 mg/mL, internal standard) were added to it. The sample was vigorously extracted for 1 min and was centrifuged at 13,000 ×g, 4°C for 10 min. 200 *μ*L of the supernatant was transferred to a GC vial and then evaporated to dryness under nitrogen at room temperature. Dried extracts were methoximated in pyridine with 50 *μ*L of 15 mg/*μ*L of methoxyamine at 30°C for 1.5 h. Metabolites were subsequently trimethylsilylated at 70°C for 1 hour with 50 *μ*L MSTFA with 1% TMCS. Finally, 40 *μ*L heptane was added to the GC vial before GC/MS analysis.

### 2.7. GC/MS Analysis

All samples were separated through an HP-5MS capillary column (30 m × 250 *μ*m id, 0.25 *μ*m film thickness, USA) and analyzed by an Agilent 6890N GC/5975B inert MSD (Agilent Technologies, Santa Clara, CA, USA). The injection and the interface temperature were set at 260°C were injected, and the ion source was adjusted to 200°C. Aliquots (1 *μ*L) were injected in the splitless mode. The following temperature program is shown in Table 1. Helium was used as the carrier gas at a flow rate of 1 mL/min. Electron energy was 70 eV, and detection was conducted in full scan mode (*m/z* 30–600). Solvent delay is 5 min.

### 2.8. Data Processing

Unprocessed GC/MS raw files were converted to NetCDF format via DataBridge (PerkinElmer Inc., USA) and subsequently processed by the XCMS toolbox (http://metlin.scripps.edu/xcms/) using XCMS default settings with the following exceptions: xcmsSet (full width at half-maximum: fwhm = 4; S/Ncutoff value: snthresh = 8, max = 20), group (bw = 10) to carry out baseline correction, peak discrimination, and alignment. The resulting table (TSV file) was exported into Microsoft Excel 2007. All data were normalized to the total sum of spectrum prior to multivariate analyses. The resulting three-dimensional matrix involving peak index (RT − *m*/*z* pair), sample names (observations), and normalized peak area were fed to SIMCA-P 11.5 software (Umetrics, Umea, Sweden) for principal component analysis (PCA), partial least squares discriminant analysis (PLS-DA), and orthogonal projections to latent structures (OPLS) after undertaking a unit variance procedure. PCA was used to exhibit the general clustering, trends, or outliers in the observations (or samples). Meanwhile, the PLS-DA model, as a method derived from PLS analysis where the *Y* matrix was set as a dummy descriptor by SIMCA-P, was used to maximize metabolite variations and identify significantly altered metabolites responsible for such variations. OPLS model was a modification of PLS, which filtered the unrelated variations, reduced model complexity, and obtained a higher level of group separation. *R* ^2^ *X* represents the cumulative modeled variation in *X*, *R* ^2^ *Y* is the cumulative modeled variation in *Y*, and *Q* ^2^ *Y* is the cumulative predicted variation in *Y*. The values of these parameters approaching 1.0 indicated a stable model with a predictive reliability. These discriminating metabolites were obtained by using a statistically significant threshold of variable influence on projection (VIP > 1.0) values obtained from the OPLS model and were further validated by Student's *t-*test. The metabolites with VIP values greater than 1.0 and *P* values <0.05 were selected as potential biomarkers between two classes of samples [20]. Additionally, potential biomarkers detected were identified by using the reference compounds available and the commercial compound libraries database NIST. The concentration of each metabolite was expressed as the ratio of its peak area value to that of the selected internal standard peak area of myristic acid. 

### 2.9. Statistics

Quantitative data was analyzed by normality test, and statistical significance among the groups was performed by the Kruskal-Wallis test. The correlation analysis was processed by pairwise Spearman between metabolite levels and clinical indicators in each group. Additionally, unpaired Student's *t*-test was utilized to evaluate the significant difference of endogenous metabolites (SPSS 17.0, Chicago, Ill, USA). The result of *P* < 0.05 was considered to be statistically significant.

## 3. Results

### 3.1. The Clinical Features of the Three Groups

Using SPSS 17.0 software, normality of the data was tested by means of the Shapiro-Wilk test, and the result showed that the data was nonnormal distribution; then, statistical significance among the groups was performed by the Kruskal-Wallis test. Clinical characteristics of three groups were shown in Table 2. Among 125 subjects, their tongue coatings revealed 50 cases with thick yellow coating, 50 cases with thick white coating, and 25 cases with normal white tongue coatings. The median age of Group A and Group B was 36 years and 37.5 years, respectively, and that of 25 healthy people was 35 years. There were no significant differences among ages. There was significant difference in Group A and Group B in distribution of gender, respectively, compared with Group C (*P* < 0.05). Levels of DBIL, AST, ALT, GGT, ALP, TBA, and HBsAg in serum were significantly increased in Group A and Group B, respectively, compared with Group C (*P* < 0.05); serum ALB content in Group A and Group B was significantly lower than that in Group C (*P* < 0.05). Other statistical significances were not found. As shown in Table 2, there were no significant changes between Group A and Group B.

### 3.2. Metabonomic Study

#### 3.2.1. GC/MS Spectra of the Three Groups

**Figure 2:**
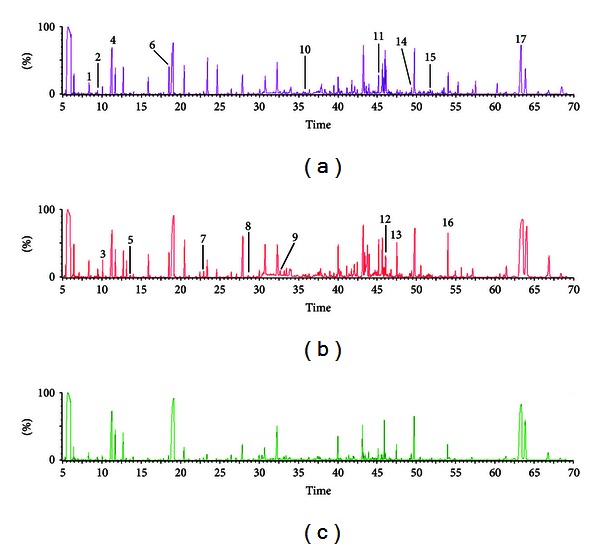
Representative GC/MS TIC chromatograms of urine samples from three groups. (a) Group A (thick yellow coating), (b) Group B (thick white coating), and (c) Group C (thin white coating). Selected significantly changed metabolites have been labeled in this study: (1) butanedioic acid, (2) propanoic acid, (3) acetic acid, (4) ethanedioic acid, (5) butanoic acid, (6) glycine, (7) 2,3-dihydroxybutanoic acid, (8) aminomalonic acid, (9) cysteine, (10) benzoic acid, (11) D-mannose, (12) sedoheptulose, (13) D-gluconic acid, (14) myo-inositol, (15) tryptophan, (16) pseudouridine, and (17) *α*-D-glucopyranoside.

Typical GC/MS total ion current (TIC) chromatograms of urine from Group A (thick yellow coating), Group B (thick white coating), and Group C (thin white coating) were shown in Figure 2. Visual inspection of these spectra showed obvious difference among the three groups. Using our optimized GC/MS analysis protocol in association with a software-based peak deconvolution procedure, the most peaks were identified as endogenous metabolites based on NIST library. The majority of these metabolites were amino acids, nucleosides, carbohydrate, and fatty acids that are mainly involved in energy metabolism, nucleoside metabolism, and amino acid metabolism.

#### 3.2.2. Analysis of Metabolic Profiles

In order to understand the general trends, differences, and outliers among three groups by GC/MS spectra, we applied a PCA and PLS-DA to the GC/MS data. The result provided unsatisfactory separation in the scores plot among three groups (data were displayed in the additional material files), probably due to the complexity of clinical samples.

**Figure 3:**
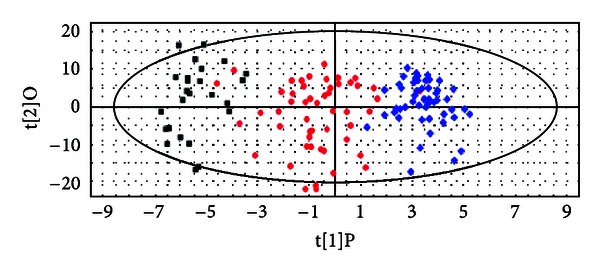
Score plot of OPLS model obtained from Group A (thick yellow coating, the blue diamond), Group B (thick white coating, the red circle), and Group C (thin white coating, the black square).

To obtain a higher level of group separation, reduce the impacts of the disturbing factors, and enhance recognition of variables responsible for classification, a supervised OPLS was applied. OPLS was a modification of PLS, which separated the systematic variation in *X* into two parts, one that is linearly related to *Y* and one that is orthogonal to *Y*, which filtered the unrelated variations, reduced model complexity with preserved prediction ability in differentiating the groups, and improved interpretative ability of variation in spectra [21, 22]. The OPLS statistical method is applied in various research areas such as public health research [23, 24]. In this study, the OPLS scores plot obtained was given in Figure 3, the *x*-axis represents the first principle component score value, *y*-axis represents the second principle score value, each point represents one sample, score plot reflects the sample distribution in the low-dimensional space after the dimensionality reduction, and each sample is represented with a point of a two-dimensional space. The points of similar samples are close to each other; the differences between samples are described in the multidimensional space. This result showed better separation than PCA and PLS-DA. The distinct clustering in metabolic profiling was observed from Group A (thick yellow coating in patients with chronic hepatitis B), Group B (thick white coating in patients with chronic hepatitis B), and Group C (thin white coating with healthy humans), suggesting that the metabolic characteristics of the three groups were distinctly different. Moreover, to estimate the robustness and the predictive ability of our model, we used 7-fold cross-validation [25]. After calculating the components for OPLS, the parameters for the classification from the software were *R* ^2^ *Y* = 0.89 and *Q* ^2^ *Y* = 0.67, respectively. High coefficient values of *R* ^2^ *Y* and *Q* ^2^ *Y* suggested that model established in this study was stable, good to interpret and predict [26]. These results demonstrated that our model was reliable.

#### 3.2.3. Metabolite Identification

**Figure 4:**
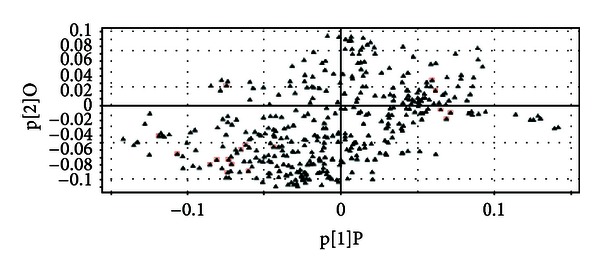
Loadings plot from OPLS model classifying model obtained from Group A, Group B, and Group C. The red square: identified metabolites.

Based on OPLS analysis, we constructed loading plot. As shown in Figure 4, the *x*-axis represents the correlation coefficient of the first principle component, the *y*-axis represents the correlation coefficient of the second principle, and each point represents a variable. The loading plot reflects the correlation between the principle components and variables, which was a visual method that can be used for selection of potential biomarkers among three groups. From the loading plot, we can know potential biomarkers that were the furthest ones from the origin, but the loading plot was complex with many variables [27].

To refine this analysis, the variable importance projection (VIP) was obtained. VIP values exceeding 1.0 were selected as potential biomarkers [28]. Furthermore, the retention times and MS/MS behaviors of metabolites with the data from databases of METLINE were compared (http://metlin.Scripps.edu/). Additionally, metabolites detected were identified by the commercial compound database NIST. Meanwhile, metabolites were searched for related pathway with available biochemical databases, such as KEGG (http://www.genome.jp/kegg/) and HMDB (http://www.hmdb.ca/). On the basis of the above analysis, finally, we generated a list of 17 variables representing individual metabolites. These metabolites were shown in Table 3.

In particular, compared with Group C (thin white coating), the concentration of glycine, cysteine, benzoic acid, D-Mannose, and tryptophan were significantly increased, and propanoic acid, ethanedioic acid, aminomalonic acid, D-gluconic acid, and *β*-D-glucopyranoside were significantly decreased in Group A (*P* < 0.05 or *P* < 0.01). In Group B (thick white coating), urine acetic acid, 2,3-dihydroxybutanoic acid, cysteine, benzoic acid, D-mannose, sedoheptulose, myo-inositol, tryptophan, and pseudouridine levels significantly increased, and levels of butanedioic acid, ethanedioic acid, and aminomalonic acid in urine were significantly decreased compared with Group C (*P* < 0.05 or *P* < 0.01), respectively. Moreover, significantly increased butanedioic acid and cysteine and significantly decreased propanoic acid, acetic acid, butanoic acid, 2,3-dihydroxybutanoic acid, D-mannose, D-gluconic acid, and myo-inositol were displayed in Group A compared with Group B (*P* < 0.05 or *P* < 0.01), which indicated that these metabolites may be possible biological markers to distinguish between these two groups.

### 3.3. Correlation Analysis of Metabolite Levels and Clinical Indicators in the Three Groups

The pairwise Spearman correlation analysis was carried out between metabolite levels and clinical indicators in each group by using SPSS 17.0 software. As were displayed in Tables 4, 5, and 6, the metabolite levels significantly (*P* < 0.05) correlated with clinical parameters were as follows: (1) butanedioic acid and TBA, 2,3-dihydroxybutanoic acid, aminomalonic acid, cysteine, benzoic acid, D-gluconic acid, *β*-D-glucopyranoside, and ALB in Group A; (2) tryptophan and ALB, TBA, ALP, ethanedioic acid and TBIL, DBIL, IDBIL, cysteine and TBIL, IDBIL, TP, *β*-D-glucopyranoside and TBIL, IDBIL, TP, benzoic acid and TBIL D-mannose, and IDBIL in Group B, (3) butanoic acid, glycine and GGT, tryptophan and ALB, acetic acid and ALT, aminomalonic acid, *β*-D-glucopyranoside, and AST in Group C. There was no significant correlation between metabolite levels and the other indicators.

## 4. Discussion

Hepatitis B virus (HBV) infection is a serious public health problem affecting more than 400 million people worldwide. Moreover, the World Health Organization estimates a death rate of 1 million people annually [29]. 2%–10% of the individuals with chronic hepatitis B (CHB) are estimated to develop liver cirrhosis, and a subset of these individuals eventually suffer from liver failure or hepatocellular carcinoma [30]. In this study, we have carried out the analysis of the clinical and biochemical indicators. A significant increase in the activities of DBIL, AST, ALT, GGT, ALP, and TBA as observed, and serum ALB content was significantly reduced in two different tongue coatings in patients with chronic hepatitis B groups compared with those of the normal control group, indicating considerable hepatocellular injury, especially, AST and ALT, is used as marker of viral hepatitis or liver damage [31]. Serum HBsAg was significantly increased, which is a specific marker of HBV infection, suggesting that patients have been infected with HBV [32]. In this study, patients with chronic hepatitis B were HBeAg positive. The HBeAg positive implied severe illness, poor prognosis, and highly infectiousness. The study showed that patients with HBeAg positive had a 2% to 4% annual incidence of cirrhosis [33]. Most patients with HBeAg seroconversion can control the progress of the disease and reduce the incidence of cirrhosis and liver cancer by effective antiviral therapy [34]. Moreover, serum HBV DNA in CHB patients was measured, result showed high level of HBV DNA. Serum HBV DNA is considered to be the most direct etiological basis of hepatitis B virus replication and infection and mainly used for the diagnosis of chronic HBV infection, treatment selection of indications, and monitoring of HBV disease both in clinical trials and in clinical practice [35–37]. In this study, patients in two abnormal tongue coatings with chronic hepatitis B were serum high level of HBsAg and HBV DNA, which indicated that the formation of pathological tongue may be related to HBV replication.

Apart from the documented clinical and biochemical indicators, urinary samples from patients with chronic hepatitis B with thick yellow coating and thick white coating, and healthy humans with thin white coating were analyzed by GC/MS. The distinct clustering between the patients and healthy humans was observed, and the samples between thick yellow coating and thick white coating in patients with chronic hepatitis B were also separated into two individual groups. Furthermore, the correlation analysis was carried out between metabolite levels and clinical indicators in each group, respectively. As was shown in Tables 4, 5, and 6, there is no significant correlation between most metabolites and clinical indicators except for minority indexes, which may illustrate little relationship between the objective material basis related to tongue coating and the clinical indicators. Our earlier study showed that the clinical indicators had limited value in determining TCM syndromes in patients with chronic hepatitis B [38]. In addition, among these clinical indicators there were no significant differences between Group A and Group B. This result reflects that the clinical indicators have limited help in terms of the tongue coating's distinguishing in TCM. As shown in Table 2, compared with Group C, levels of some clinical indicators in serum were significantly changed in Group A and Group B, respectively, and obvious alteration of these indicators may be caused by the disease; these indicators contributed to the diagnosis of the disease. The above data further implied that reductionism was difficult to reflect the essence of Chinese medicine; the holistic application of metabonomics may be more suitable for studying the differentiation of the tongue coating in TCM.

In this study, we constructed a PCA and PLS-DA model to obtain metabolic profiling. The samples among three groups were not clearly separated (data were displayed in the additional material files). The *R* ^2^ *X*  and *Q* ^2^ *X* of the former model were 0.48 and 0.39, whereas the *R* ^2^ *Y* and *Q* ^2^ *Y* of the latter model were 0.58 and 0.35. These results indicated that the models had a poor ability of explaining and predicting. Reasons may be that humans are complex and diverse organisms, and the clinical samples are susceptible to a variety of factors, including diet, age, lifestyle, gender, and many more factors [39, 40]. In order to obtain a higher level of group separation and reduce the impacts of the disturbing factors, we applied a supervised OPLS, which filtered the unrelated variations, and obtained a higher level of group separation among the three groups. The *R* ^2^ *Y* and *Q* ^2^ *Y* of the model were 0.89 and 0.67. These parameters for the classification indicated that the model had a good ability of explaining and predicting. In addition, there were obvious differences among endogenous metabolites in the three groups, which may suggest that metabolic pathways related with two different tongue coatings in patients have changed.

As shown in Table 3, compared with Group C, the concentration of D-mannose was significantly increased, and propanoic acid, D-gluconic acid, and *β*-D-glucopyranoside were significantly decreased in Group A (*P* < 0.05 or *P* < 0.01). In Group B, urine D-mannose, myo-inositol, and sedoheptulose levels significantly increased, and levels of butanedioic acid and ethanedioic acid in urine were significantly decreased compared with Group C (*P* < 0.05 or *P* < 0.01), respectively. The above results indicated that objective material basis of the tongue coating may be associated with energy metabolism. Propionic acid is the main source for glucose production [41]. Butanedioic acid and ethanedioic acid are the intermediates of tricarboxylic acid (TCA) cycle and provide an easy energy supply for the body. D-Gluconic acid is involved in pentose phosphate pathway. Sucrose is synthesized by *β*-D-glucopyranoside and D-glucose and participates in energy metabolism. D-Mannose is a monosaccharide, forms mannose 6-phosphate, and enters glycolysis with the role of hexokinase phosphorylation in carbohydrate metabolism process. Myo-inositol is a necessary nutrient source for mammals and participated in the inositol phosphate metabolism and provided energy for the body. Sedoheptulose is produced from the oxidative pentose phosphate pathway intermediate sedoheptulose-7-phosphate, by a sedoheptulose-7-phosphate phosphatase [42]. The liver is the central organ of the metabolism of nutrients; liver damage that is caused by any reason will lead to abnormality of material and energy metabolism, which resulted in varying degrees of malnutrition and adversely affected patients well-being and survival [43, 44]. According to Chinese medicine theory, “The spleen governs transportation and transformation, the spleen transports and transforms food nutrients and water, are distributed to the whole body and nourished the four limbs and the other parts of the body,” and it is said that “the spleen is the acquired base of life” and “the source of qi, blood and body fluid” [45]. This process corresponds to the energy circulating in the human body. The above results showed that energy metabolism may be disturbed in the formation of the pathological tongue coating.

We can see from Table 3 that the levels of glycine, cysteine, and tryptophan were significantly increased in Group A, and the levels of cysteine and tryptophan were significantly increased in Group B compared with Group C (*P* < 0.05), respectively, which suggested the abnormality of amino acid metabolism. Glycine exerts anti-inflammatory, cytoprotective, and immunomodulatory properties and ameliorates liver injury through attenuation of oxidative stress, apoptosis [46]. Homocysteine (HCY) is a sulfur-containing amino acids, mainly derived from S-adenosyl homocysteine generated by S-adenosylmethionine methyltransferase, and mainly completed catabolism in the liver [47]. When metabolism is impaired, HCY accumulation occurred within the cell, leading to increase HCY content and the burden of the liver cells, which brought about the metabolism barrier of amino acids and HCY [48]. Tryptophan is an essential amino acid that maintains normal cell metabolism and proliferation in the body, and it is also an important nutrient and involved in protein synthesis [49]. When liver cells are damaged, the release of free tryptophan is increased; thus, liver disease can significantly influence the metabolic state of tryptophan in the body [50]. It was believed that changes of tongue coating were mainly correlated with the exogenous pathogenic factors and the nature of disease [51]. When the body's normal metabolism was disordered, pathological tongue coating would appear. In this study, two different tongue coatings with chronic hepatitis B patients showed abnormality of amino acid metabolism; we could infer that amino acid metabolism is possiblily disturbed in the pathological tongue coating formation process. 

The changes of benzoic acid, butanoic acid, acetic acid, and 2,3-dihydroxybutanoic acid are associated with gut microflora. Benzoic acid is the metabolite of gut microflora, and its metabolism parent is mainly polyphenols taken from food and aromatic amino acids obtained from food protein decomposition [52]. Butanoic acid, acetic acid, and 2,3-dihydroxybutanoic acid are short-chain fatty acids mainly generated by dietary cellulose, starch, and other undigested substances under microbial fermentation in the cecum or colon [53]. The human gut microbiome plays an important role in maintaining natural host-environment interactions involved in nutrient absorption, epithelium regeneration, energy metabolism, and immune response [54]. It was reported that the tongue shares considerable similarity with the gut not only in mucosal epithelia but also microbial diversity [55, 56]. Tongue is consists of the mucous layer, lamina propria, and muscularis; the formation of the tongue coating may be related to metabolism process of keratinized epithelial cells of the mucous layer [57]. A large number of bacteria, exfoliated epithelial cells of the oral mucosa, and food residues in the tongue coating are a good living environment in the oral microbial [58]. Many bacteria species have been found in tongue coating [9]. The normal flora in tongue coating stimulates the host immune system, which is conducive to the health of the host; the host provides a suitable environment and nutrition for microorganisms, and a dynamic balance is maintained between the two. Under abnormal conditions, host nutritional intake, immunity, disease, and drugs caused the abnormal appearances of the tongue coating; imbalanced microecology in tongue coating appeared, leading to alterations of the number and types of various microorganisms, and the emergence of pathological tongue coating [59, 60]. Therefore, the pathological changes in tongue coating were associated with the abnormal changes of microecological environment in tongue coating. It is recently reported that the bacteria species may have a close correlation with the formation of greasy fur of chronic gastritis; the microbial changes in the oral cavity may be one of the formation mechanisms for greasy tongue coating [61]. Kazor et al. compared the microbial profiles of the tongue dorsa of healthy subjects and subjects with halitosis; the result displayed that the predominant microbiota on the tongue dorsa of healthy subjects was different from that on the tongue dorsa of subjects with halitosis [62]. In this study, from the perspective of the metabolite levels, the alteration of these metabolites may indicate that the structure or activity of intestinal symbiotic bacteria has been greatly disturbed by the viruses that may cause the formation of pathological tongue coating.

Another result showed that pseudouridine was significantly increased in Group B compared with Group C, which suggested the dysfunction of nucleotide metabolism. It is known that pseudouridine originating from RNA degradation is excreted in abnormal levels in the urine of patients with malignant tumour, and they have been proposed as tumour markers for the early diagnosis of the tumor, the differential diagnosis of tumor progression and recurrence monitoring, and efficacy and prognosis judgment [63]. In Group B, the level of pseudouridine was significantly increased; therefore, we speculated that change of pseudouridine in urine may be related to the abnormality of thick white coating.

In addition, the level of aminomalonic acid was significantly decreased in Group A and Group B compared with Group C, respectively. Its biological connotations related to the tongue coating remain unclear and will be followed up by further studies in our laboratory.

 The common feature of the two different tongue coatings in patients with chronic hepatitis B involves abnormality of energy metabolism, amino acid metabolism; intestinal flora metabolism, and other metabolic pathways, which are the results of the changes of multiple system functions in the body, and there are different metabolic characteristics between the two different tongue coatings. Significant differences are displayed in the level of butanedioic acid, propanoic acid, acetic acid, butanoic acid, 2,3-dihydroxybutanoic acid, cysteine, D-mannose, D-gluconic acid, and myo-inositol between the two different tongue coatings; these metabolites may be the material basis of the two tongue coatings differentiation.

## 5. Conclusions

In this paper, we studied on urine metabolic profiles of two different tongue coatings in patients with chronic hepatitis B using GC/MS based on metabonomic method. Multivariate statistical analysis showed a good separation among two different coating groups and healthy control group. At the same time, 17 significantly changed metabolites have been found and identified, which indicated that the material basis leading to the formation of the tongue coating was related to energy metabolism, amino acid metabolism, nucleotide metabolism, and gut microflora. The results of biological parameters and the metabonomic techniques suggested that the tongue coating may have the objective material basis, which has little relation to the clinical indicators. This work also indicated that some significantly changed metabolites may be potential biomarkers to distinguish between two different tongue coatings in patients with chronic hepatitis B. However, limited to the time and cost, we studied only samples of urine of two different tongue coatings in patients with chronic hepatitis B. Further research is planned to study on the tongue coating and serum metabolic profiles of the different tongue coatings in patients with chronic hepatitis B and other diseases in order to well explain the objective material basis leading to the formation of tongue coating.

## Figures and Tables

**Table 1:** The temperature program of GC/MS.

Heating rate (°C/min)	Temperature (°C)	Holding time (min)
	70	2
2.5	160	0
5	240	16

**Table 2:** Clinical and biological characteristics of 100 patients with chronic hepatitis B and 25 normal subjects.

	Group A	Group B	Group C
	(*n* = 50)	(*n* = 50)	(*n* = 25)
Age (years)	36 (22, 58)	37.5 (17, 50)	35 (20, 57)
Gender (M/F)	50 (43/7)*	50 (44/6)*	25 (15/10)
Course of disease (years)	4 (0.6, 23)	7.5 (0.6, 27)	—
TBIL (*μ*mol/L)	14.21 (5.2, 81.21)	18.19 (8.65, 245.41)	14.9 (6.9, 20.5)
DBIL (*μ*mol/L)	5.75 (2.5, 62.9)*	6.35 (2.5, 201.4)*	4.2 (3, 5.3)
IDBIL (*μ*mol/L)	10.15 (1.3, 28.7)	11.75 (4.5, 44)	10.7 (3.9, 15.9)
ALT (IU/L)	66 (23, 365)*	67.5 (22, 584)*	17 (10, 31)
AST (IU/L)	39.5 (25, 131)*	37.5 (20, 286)*	19 (14, 35)
GGT (IU/L)	39.5 (7, 436)*	45 (5, 634)*	19 (10, 63)
ALP (IU/L)	84 (47, 211)*	96 (39, 182)*	53 (13, 96)
TP (g/L)	73.5 (53, 86)	74 (61, 84)	72 (62, 79)
ALB (g/L)	30 (9, 48)*	28 (19, 49)*	44 (38, 63)
TBA (*μ*mol/L)	16 (2, 311)*	17.5 (2, 390)*	8 (5, 12)
HBsAg (IU/mL)	250 (15.25, 250)*	250 (0.27, 250)*	0.01 (0, 0.02)
HBeAg (S/CO)	37 (13)	30 (20)	—
log DNA (copies/mL)	5.03 (3.05, 9.24)	5.01 (3.13, 8.04)	—

Statistical analysis of data among groups was performed by the Kruskal-Wallis test, and significant differences were compared with Group C (*P < 0.05).

**Table 3:** Summary of metabolites revealed in this study.

Metabolites	Group A versus Group C (fold change)	*P* value	Group B versus Group C (fold change)	*P* value	Group A versus Group B (fold change)	*P* value	Pathways
Butanedioic acid	+1.15	NS	−1.86*	0.043	+2.13^##^	0.008	Energy metabolism
Propanoic acid	−2.20*	0.02	−1.11	NS	−1.97^#^	0.025	Energy metabolism
Acetic acid	−1.02	NS	+1.65*	0.04	−1.68^##^	0.004	Flora metabolism
Ethanedioic acid	−2.24*	0.014	−2.32**	0.003	+1.03	NS	Energy metabolism
Butanoic acid	−1.18	NS	+1.36	NS	−1.60^#^	0.02	Flora metabolism
Glycine	+1.97*	0.039	+1.55	NS	+1.27	NS	Amino acid metabolism
2,3-Dihydroxybutanoic acid	+1.23	NS	+2.27*	0.047	−1.85^#^	0.032	Flora metabolism
Aminomalonic acid	−3.21**	0.002	−2.16*	0.017	+1.49	NS	Unknown
Cysteine	+2.11*	0.028	+2.40*	NS	+1.79^#^	0.04	Amino acid metabolism
Benzoic acid	+2.12*	0.016	+1.80*	0.029	−1.1	NS	Flora metabolism
D-Mannose	+1.71*	0.042	+1.48**	0.006	−1.93^#^	0.011	Energy metabolism
Sedoheptulose	+1.11	NS	+3.30*	0.038	−1.33	NS	Energy metabolism
D-Gluconic acid	−1.84*	0.016	+1.00	NS	−1.84^#^	0.01	Energy metabolism
Myo-inositol	−1.08	NS	+1.79*	0.042	−1.93^##^	0.0045	Energy metabolism
Tryptophan	+2.08*	0.016	+2.05*	0.035	+1.01	NS	Amino acid metabolism
Pseudouridine	+1.43	NS	+1.89*	0.033	−1.32	NS	Nucleotide metabolism
*α*-D-Glucopyranoside	−2.84*	0.033	−1.61	NS	−1.76	NS	Energy metabolism

“+” and “−” represent the relative increased (or decreased) concentration of metabolites; fold changes were obtained by calculating the relative concentration between the two group; *P < 0.05, **P < 0.01 compared with Group C (thin white coating); ^##^ *P* < 0.01, ^#^ *P* < 0.05 compared between Group A (thick yellow coating) and Group B (thick white coating); NS indicates nonsignificant; statistical significance (*P* < 0.05, unpaired Student's *t*-test).

**Table 4:** Correlation analysis of metabolite levels and clinical indicators in Group A.

	TBIL	DBIL	IDBIL	ALT	AST	GGT	ALP	TP	ALB	TBA
Butanedioic acid	0.042	0.029	0.015	0.075	−0.118	−0.064	−0.089	0.171	−0.051	−0.410**
Propanoic acid	−0.054	−0.105	−0.056	0.131	0.109	0.225	−0.079	−0.160	−0.176	0.053
Acetic acid	−0.085	−0.088	−0.123	0.194	0.128	0.195	−0.104	−0.046	−0.251	−0.006
Ethanedioic acid	0.228	0.111	0.229	0.012	−0.063	0.016	0.131	0.097	−0.219	−0.169
Butanoic acid	−0.014	−0.036	−0.066	0.122	0.092	0.047	0.094	0.012	−0.187	−0.039
Glycine	−0.186	−0.229	−0.173	0.183	0.059	0.059	−0.159	−0.014	−0.179	−0.234
2,3-Dihydroxybutanoic acid	0.045	0.036	−0.006	0.100	0.043	0.045	−0.004	−0.002	−0.282*	−0.111
Aminomalonic acid	0.011	−0.029	−0.017	0.088	0.090	0.176	−0.027	−0.062	−0.299*	0.067
Cysteine	0.032	−0.027	0.017	0.023	0.005	0.115	0.122	−0.048	−0.370**	−0.060
Benzoic acid	0.100	0.076	0.089	0.027	−0.003	0.064	0.029	−0.239	−0.426**	0.089
D-Mannose	−0.106	−0.130	−0.109	0.189	0.160	0.154	−0.112	−0.115	−0.162	−0.047
Sedoheptulose	−0.150	−0.178	−0.126	0.141	0.050	0.124	−0.145	−0.097	−0.145	−0.072
D-Gluconic acid	0.181	0.123	0.125	−0.002	−0.100	−0.007	0.136	0.181	−0.337*	0.076
Myo-inositol	−0.206	−0.195	−0.238	0.135	0.110	0.234	−0.228	−0.048	0.031	−0.089
Tryptophan	−0.087	−0.018	−0.082	0.258	0.073	−0.015	−0.057	−0.012	0.097	−0.163
Pseudouridine	0.062	0.043	0.066	0.008	−0.193	−0.168	−0.012	0.204	−0.129	−0.460
*β*-D-Glucopyranoside	−0.026	0.011	0.011	−0.128	−0.073	−0.003	0.005	−0.102	0.357*	−0.137

*Correlation is significant at the 0.05 level (2-tailed).

**Correlation is significant at the 0.01 (2-tailed).

**Table 5:** Correlation analysis of metabolite levels and clinical indicators in Group B.

	TBIL	DBIL	IDBIL	ALT	AST	GGT	ALP	TP	ALB	TBA
Butanedioic acid	0.053	−0.001	0.020	0.047	−0.063	−0.074	0.074	0.207	−0.031	0.199
Propanoic acid	0.194	0.183	0.112	0.160	0.189	−0.092	0.099	−0.095	0.031	−0.068
Acetic acid	0.100	−0.011	0.115	0.092	0.080	−0.130	−0.017	0.133	0.061	−0.123
Ethanedioic acid	0.469**	0.318*	0.451**	0.208	0.046	0.020	0.164	0.217	−0.200	0.037
Butanoic acid	0.062	−0.010	0.122	0.224	0.106	−0.102	0.021	0.156	0.023	0.020
Glycine	0.179	0.081	0.125	0.060	−0.005	−0.015	0.201	0.194	−0.089	0.170
2,3-Dihydroxybutanoic acid	0.168	−0.011	0.239	0.012	−0.060	−0.055	0.038	0.249	−0.070	0.076
Aminomalonic acid	0.045	0.014	0.075	0.032	−0.094	−0.183	0.031	0.140	0.097	−0.092
Cysteine	0.300*	0.168	0.388**	0.099	0.067	0.089	0.083	0.294*	−0.054	0.045
Benzoic acid	0.279*	0.128	0.276	0.153	0.054	−0.120	0.118	0.210	−0.072	0.005
D-Mannose	0.204	0.045	0.282*	0.174	0.151	−0.017	−0.040	0.204	0.144	−0.051
Sedoheptulose	0.174	0.081	0.180	0.157	0.042	−0.049	0.163	0.003	0.020	0.061
D-Gluconic acid	−0.066	−0.127	0.042	0.130	0.068	−0.141	−0.087	−0.034	0.130	−0.122
Myo-inositol	0.158	0.025	0.185	−0.004	0.042	−0.089	0.045	0.036	0.142	−0.045
Tryptophan	0.082	0.057	−0.001	0.183	0.264	0.139	0.338*	0.062	−0.301*	0.287*
Pseudouridine	0.024	−0.086	0.052	0.160	0.031	−0.129	−0.214	0.219	0.068	−0.042
*β*-D-Glucopyranoside	0.432**	0.259	0.445**	0.011	0.074	0.118	0.000	0.285*	0.109	−0.067

*Correlation is significant at the 0.05 level (2-tailed).

**Correlation is significant at the 0.01 (2-tailed).

**Table 6:** Correlation analysis of metabolite levels and clinical indicators in Group C.

	TBIL	DBIL	IDBIL	ALT	AST	GGT	ALP	TP	ALB	TBA
Butanedioic acid	0.026	0.261	0.022	−0.110	0.149	−0.231	0.161	0.162	0.148	0.278
Propanoic acid	−0.135	0.047	−0.107	−0.014	0.213	−0.281	0.149	0.302	−0.071	−0.023
Acetic acid	−0.078	−0.160	−0.022	−0.518**	−0.367	−0.315	−0.111	0.110	−0.098	0.219
Ethanedioic acid	0.120	0.082	0.123	0.048	−0.279	0.003	0.171	−0.069	−0.295	−0.210
Butanoic acid	0.077	−0.002	0.142	−0.170	−0.013	−0.483*	−0.055	0.018	−0.229	0.094
Glycine	0.148	0.328	0.159	−0.031	0.183	−0.428*	0.148	0.073	0.006	0.283
2,3-Dihydroxybutanoic acid	−0.280	−0.048	−0.244	−0.164	−0.170	−0.167	−0.006	0.061	−0.205	0.208
Aminomalonic acid	0.349	0.049	0.341	−0.184	−0.447*	−0.053	−0.124	−0.010	−0.004	−0.331
Cysteine	−0.168	0.180	−0.159	−0.125	0.233	−0.341	0.238	0.237	0.033	0.308
Benzoic acid	0.188	0.276	0.182	−0.143	−0.106	−0.277	0.022	0.033	0.047	−0.060
D-Mannose	−0.046	−0.126	0.009	−0.265	−0.130	−0.332	0.034	0.173	−0.079	0.127
Sedoheptulose	−0.236	−0.157	−0.235	0.102	0.083	−0.095	0.186	0.192	0.086	0.125
D-Gluconic acid	−0.043	−0.298	−0.042	0.066	0.278	−0.037	−0.070	0.094	−0.078	−0.051
Myo-inositol	0.080	0.017	0.108	0.166	0.181	−0.323	0.214	0.046	−0.164	−0.049
Tryptophan	−0.212	−0.128	−0.207	−0.119	0.066	−0.108	0.322	−0.155	−0.462*	−0.337
Pseudouridine	−0.185	−0.020	−0.157	−0.159	0.120	−0.215	0.071	0.337	0.104	0.326
*β*-D-Glucopyranoside	−0.241	0.036	−0.237	0.205	0.486*	−0.364	0.290	0.135	−0.191	0.272

*Correlation is significant at the 0.05 level (2-tailed).

**Correlation is significant at the 0.01 (2-tailed).
